# PPARγ Ligands Attenuate Hypoxia-Induced Proliferation in Human Pulmonary Artery Smooth Muscle Cells through Modulation of MicroRNA-21

**DOI:** 10.1371/journal.pone.0133391

**Published:** 2015-07-24

**Authors:** David E. Green, Tamara C. Murphy, Bum-Yong Kang, Charles D. Searles, C. Michael Hart

**Affiliations:** 1 Department of Medicine, Division of Pulmonary, Allergy, and Critical Care Medicine, Atlanta Veterans Affairs Medical Center / Emory University, Atlanta, GA, United States of America; 2 Department of Medicine, Division of Cardiology, Atlanta Veterans Affairs Medical Center / Emory University, Atlanta, GA, United States of America; Vanderbilt University Medical Center, UNITED STATES

## Abstract

Pulmonary hypertension (PH) is a progressive and often fatal disorder whose pathogenesis involves pulmonary artery smooth muscle cell (PASMC) proliferation. Although modern PH therapies have significantly improved survival, continued progress rests on the discovery of novel therapies and molecular targets. MicroRNA (miR)-21 has emerged as an important non-coding RNA that contributes to PH pathogenesis by enhancing vascular cell proliferation, however little is known about available therapies that modulate its expression. We previously demonstrated that peroxisome proliferator-activated receptor gamma (PPARγ) agonists attenuated hypoxia-induced HPASMC proliferation, vascular remodeling and PH through pleiotropic actions on multiple targets, including transforming growth factor (TGF)-β1 and phosphatase and tensin homolog deleted on chromosome 10 (PTEN). PTEN is a validated target of miR-21. We therefore hypothesized that antiproliferative effects conferred by PPARγ activation are mediated through inhibition of hypoxia-induced miR-21 expression. Human PASMC monolayers were exposed to hypoxia then treated with the PPARγ agonist, rosiglitazone (RSG,10 μM), or in parallel, C57Bl/6J mice were exposed to hypoxia then treated with RSG. RSG attenuated hypoxic increases in miR-21 expression in vitro and in vivo and abrogated reductions in PTEN and PASMC proliferation. Antiproliferative effects of RSG were lost following siRNA-mediated PTEN depletion. Furthermore, miR-21 mimic decreased PTEN and stimulated PASMC proliferation, whereas miR-21 inhibition increased PTEN and attenuated hypoxia-induced HPASMC proliferation. Collectively, these results demonstrate that PPARγ ligands regulate proliferative responses to hypoxia by preventing hypoxic increases in miR-21 and reductions in PTEN. These findings further clarify molecular mechanisms that support targeting PPARγ to attenuate pathogenic derangements in PH.

## Introduction

Pulmonary hypertension (PH) is a progressive disorder associated with significant morbidity and mortality. The pathobiology of PH is complex, and factors that contribute to vascular smooth muscle cell (SMC) proliferation play a central role in disease pathogenesis [1]. Among these factors, hypoxia is a potent stimulus associated with enhanced proliferation of human pulmonary artery smooth muscle cells (HPASMC) [2]. Despite advances in current therapies that target vasoconstriction in PH, including prostacyclin derivatives, endothelin receptor antagonists, and phosphodiesterase type 5 inhibitors, the morbidity and mortality related to PH remains high, indicating the need for novel therapeutic approaches.

Targeting the nuclear hormone receptor, peroxisome proliferator-activated receptor gamma (PPARγ) with pharmacological ligands such as the antidiabetic drug, rosiglitazone (RSG), represents a novel therapeutic strategy with diverse cellular and hemodynamic effects[3, 4]. Our lab and others demonstrated that activation of PPARγ attenuated PH and vascular remodeling in experimental animal models[5–12]. Conversely, loss of PPARγ function is associated with PH, and PPARγ expression is reduced in the lungs and pulmonary vascular tissue of patients with PH, and in experimental models of PH[2, 6, 8, 13–18]. The importance of PPARγ in vascular SMC was illustrated by Hansmann and colleagues who found that mice lacking PPARγ in the SMC compartment spontaneously developed PH[15]. Consistent with this finding, in vitro PPARγ depletion enhanced SMC proliferation[19]. Collectively, these studies highlight the importance of PPARγ as a central antiproliferative mediator and regulator of vascular homeostasis in PH.

Several publications demonstrate that PPARγ agonists confer therapeutic effects in PH by modulating the imbalanced expression of several cellular mediators of PH, including apelin[20], endothelin-1[21], Nox4 [2, 5, 16, 22], thrombospondin-1[6], NFκB[2, 16, 19], eNOS[23], TGF-β1[15] and phosphatase and tensin homolog deleted on chromosome 10 (PTEN)[6]. PTEN is a dual specificity phosphatase which exerts major antiproliferative effects on multiple cell types by inhibiting tyrosine kinase and PI3 kinase signaling in target cells[24, 25]. Accumulating evidence indicates that PH is associated with reduced expression of PTEN[6, 26, 27]. Our group found that RSG both prevented and reversed established PH in mice and blunted hypoxia-induced reductions in lung PTEN levels[6]. The current study extends these findings in the mouse lung and examines the mechanism by which PPARγ ligands modulate PTEN in HPASMC. Specific mechanisms by which PTEN expression is regulated in PH are not completely understood, but may involve post-transcriptional inhibition of PTEN gene expression by the small non-coding RNA, microRNA (miR)-21.

MiRs fine-tune gene expression by binding to target mRNAs, a process that leads to mRNA degradation or inhibition of protein translation. Several studies suggest that miR-21, which is increased in the lung in response to hypoxia [28–32] and TGF-β1[29, 30, 33] plays a central role in PH pathogenesis[29, 31, 34, 35] by enhancing SMC proliferation[31, 34, 36] and migration[34]. Since PTEN is a known target of miR-21 [28, 36, 37], we hypothesized that hypoxic increases in miR-21 suppress PTEN and promote PASMC proliferation and that PPARγ activation, by attenuating hypoxia-induced increases in miR-21 expression, reduces proliferation. Although miR-21 has been demonstrated to be involved in proliferation of VSMC in PH[31, 34], the link between PPARγ and miR-21 is not known. The current findings further clarify posttranscriptional mechanisms of gene regulation that contribute to HPASMC proliferation and define additional mechanisms of action for the therapeutic effects of PPARγ agonists in PH.

## Materials and Methods

### Mouse model of chronic hypoxia-induced PH

All animals used in the current study were male C57/BL6J mice obtained from Jackson Laboratories. Mice were 8–10 weeks old and weighed approximately 25 grams at study onset. All animals were SPF and drug-naïve. Animals were randomly allocated to 4 experimental groups that contained 3–5 mice per group. Mice were housed in cages at room air (21% oxygen) or in a hypoxia chamber for three weeks where they breathed air containing 10% oxygen to simulate the conditions of oxidative stress that promote pulmonary hypertension. Mice in the control group were exposed to normoxia and treated with vehicle, methylcellulose. In the remaining experimental groups, mice were exposed to normoxia + RSG (Cayman Chemical Company, Ann Arbor, MI) or hypoxia + RSG or methylcellulose. Drug intervention with the PPARγ activator, RSG (10 mg/kg, 100 μl) or an equal volume of methylcellulose was administered daily via oral gavage for the last 10 days of the study period. This dose was chosen because previous studies from our lab demonstrated that hypoxia-induced pulmonary hypertension, right ventricular hypertrophy and vascular remodeling were attenuated at this drug dose [5, 6]. Oral gavage was chosen as the preferred method of medication delivery because it is a minimally invasive technique that does not cause undue pain or distress. At the conclusion of the study, mice were euthanized by insufflating CO_2_ from a compressed gas tank into a clear plastic chamber in which they were housed for 3–4 minutes. Death was primarily verified by detecting a prolonged absence of spontaneous respirations, and secondarily by thoracotomy with direct visualization and removal of the heart and lungs. Protein, mRNA and microRNA were then extracted from homogenized lung tissue. All animals had access to standard mouse chow and water *ad libitum*. All scientific investigations and procedures that involved animals were reviewed and formally approved by the Atlanta Veterans Affairs Medical Center Institutional Animal Care and Use Committee.

### Cell Culture and in vitro hypoxia studies

Monolayers of HPASMC (Lonza, Walkersville, MD) or (ScienCell Carlsbad, CA) were incubated at 37°C in complete smooth muscle growth medium. In selected studies, HPASMC monolayers were placed in a normobaric hypoxia chamber (1% O_2_, 5% CO_2,_ Biospherix, Lacuna, NY) or propagated in a cell culture incubator under normoxic conditions (21% O_2_, 5% CO_2_) for 24–72 hours. RSG (10 μM in vehicle) or an equal volume of vehicle (1% dimethyl sulfoxide, Fisher Scientific, Fair Lawn, NJ) was added to the HPASMC culture media during the final 24 hours of exposure to normoxic or hypoxic conditions. In selected studies that assessed the effect of TGF-β1 inhibition on miR-21 expression, neutralizing antibodies to TGF-β1 (1 μg/ml) (R & D Systems, Minneapolis, MN) were applied to HPASMC monolayers for four hours preceding placement into the hypoxia chamber.

### Assays of HPASMC proliferation

Equal numbers of HPASMC were plated on 12- or 96-well plates and subjected to specified treatment conditions. HPASMC proliferation was determined using 3-(4,5-dimethylthiazol-2-yl)-2,5-diphenyltetrazolium bromide assay (MTT; ATCC, Manassas, VA) as we recently reported[22]. In complimentary studies, HPASMC were plated in equal numbers on 12-well plates, and proliferation was measured following experimental treatment by manual cell counting using a standard hemocytometer.

### RNA and microRNA isolation, reverse transcription and quantitative PCR

The mirVana miRNA Isolation Kit was used to extract and purify large RNA and small (micro) RNA that were enriched from HPASMC monolayer lysates or mouse lung tissue homogenates according to the manufacturer’s protocol (Life technologies, Grand Island, NY). In brief, following organic extraction and purification procedures, both large and small RNA fractions were eluted in separate vials, and RNA concentration and purity were determined by ultraviolet absorption using a spectrophotometer (BioTek, Winooski, VT). Complementary DNA (cDNA) was generated from 125–250 ng RNA per sample using the miScript II RT kit (Qiagen, Valencia, CA), and qRT-PCR was conducted with the 7500 Fast Real-Time PCR (Applied Biosystems Foster City, CA) using the primer sequences below. The amplified miR gene product was detected with QuantiTect SYBR Green PCR Kit (Qiagen). In parallel extractions, large RNA species were isolated and purified from HPASMC monolayer lysates or lung tissue homogenates using the RNeasy Mini Kit (Qiagen) as recently described[22]. In each sample, expression of target miRs and large RNA were normalized to the respective endogenous content of reference genes RNU6B, or 9S. The relative abundance of target mRNA in each sample was calculated using the ΔΔCt method[38]. Primer sequences were as follows: hsa-miR-21-5p UAGCUUAUCAGACUGAUGUUGA, Qiagen); PTEN forward (AGACCATAACCCACCACAGC), reverse (TTACACCAGTCCGTCCTTTCC); TGF-β1 forward (GCAGCACGTGGAGCTGTA), reverse (CAGCCGGTTGCTGAGGTA); 9S forward (CTGACGCTTGATGAGAAHHAC), reverse (CAGCTTCATCTTGCCCTCAT); (Eurofins MWG Operon, Huntsville, AL).

### HPASMC transfection

Lipofectamine RNAiMAX Reagent was employed in experiments that examined cellular effects of miR-21 mimic or locked nucleic acid miR-21 inhibition according to the manufacturer’s protocol (Life technologies). Dicer-substrate RNAi methods using TriFecta (IDT, Coralville, Iowa) were employed in experiments that examined cellular effects of PTEN depletion. Efficacy of mRNA or protein knockdown was determined post transfection. Small interfering RNA construct sequences that were employed include (5’ to 3’): hsa-antimiR-21 miRCURY LNA (CAACATCAGTCTGATAAGCT) (Exiqon, Woburn, MA); negative Control A miRCURY LNA (TAACACGTCTATACGCCCA) (Exiqon); hsa-miR-21-5p mature miRNA-21 mimic (UAGCUUAUCAGACUGAUGUUGA) (Ambion Life technologies); siPTEN (GCAGCUUACAUGUCUGAAGUUACTT) (TriFecta, IDT); All stars negative control siRNA (Qiagen).

### Western Blot analysis

After treatment, HPASMC monolayers were washed, scraped, and lysed. Protein was then subjected to Western blot analysis as previously reported[22]. Rabbit monoclonal primary antibodies to human PTEN (1:1000 dilution, Abcam, Cambridge, MA) were targeted by horseradish peroxidase-conjugated secondary antibodies (Jackson ImmunoResearch, West Grove, PA), or infrared dye-based secondary antibodies (Li-Cor Biotechnology, Lincoln, NE). Immunodetection was then performed using chemiluminescence (SuperSignal, Pierce Biotechnology, Rockford, IL) or infrared dye imaging (Li-Cor). Relative protein levels were quantified using the Chemidoc XRS imaging system and Quantity One software (Bio-Rad Laboratories) or Li-Cor proprietary software. Samples were normalized to their respective content of cyclin-dependent kinase (CDK 4–1:1,000 dilution, Santa Cruz Biotechnology, Dallas, TX) generated from rabbit polyclonal antibodies to mouse host antigens.

### Statistical analysis

A one-way ANOVA and an alpha of 0.05 was used to determine overall differences between sub-groups. In groups that significantly differed, all pairwise comparisons were tested using Tukey’s method for multiple comparisons to determine the nature of this difference. The plan for statistical analysis assumed normal distribution of data. Differences in groups featuring experiments with 2 variables were detected using 2-tailed unpaired t-tests.

## Results

### Hypoxia-induced reductions in PTEN expression are attenuated by the administration of RSG

**Fig 1:**
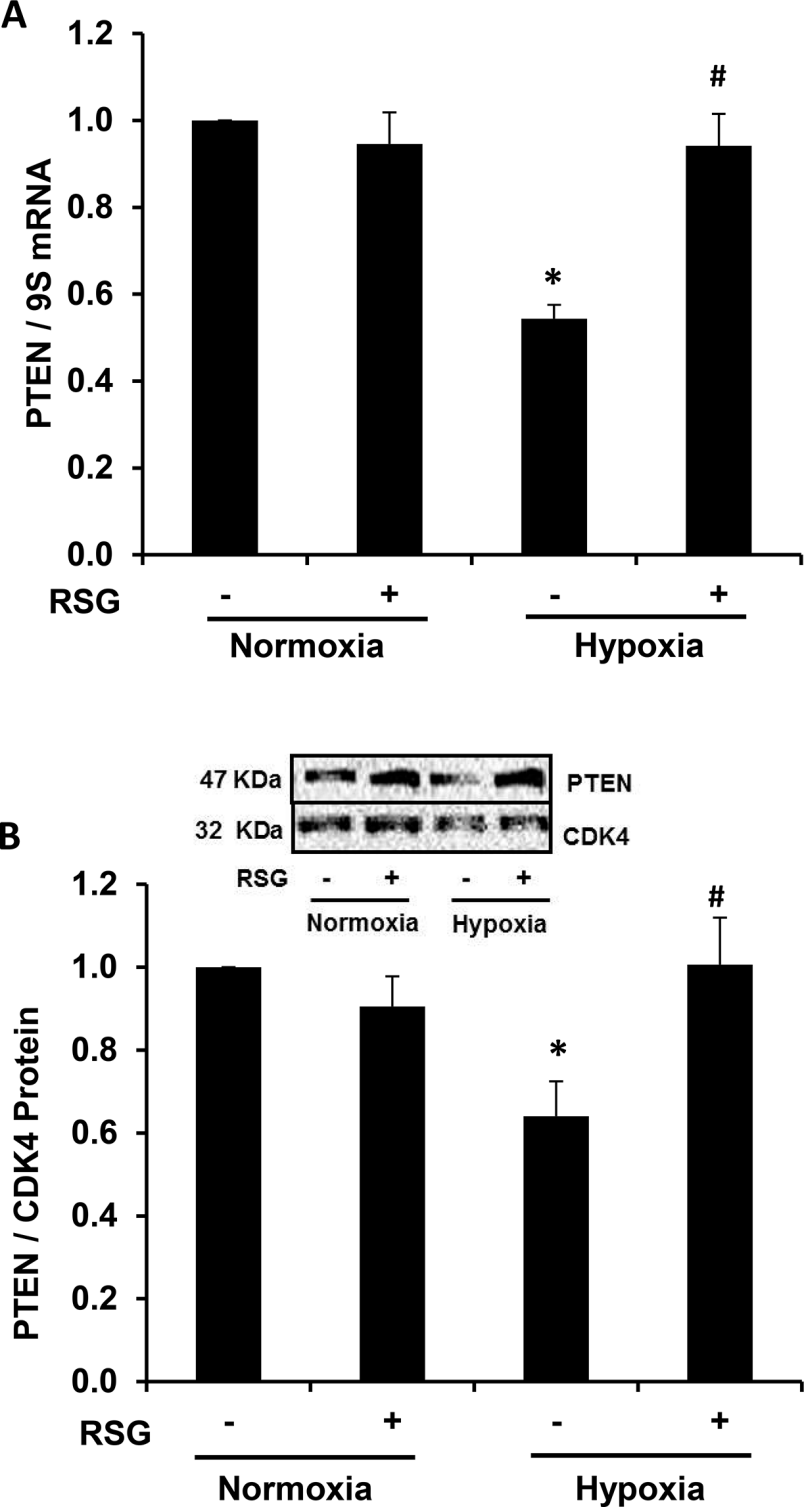
Rosiglitazone attenuates hypoxia-induced reductions in HPASMC PTEN expression. HPASMC were exposed to hypoxic conditions for 72 hours, and RSG was administered to selected monolayers during the last 24 hours of exposure. PTEN mRNA and protein levels were determined in all groups. A representative immunoblot is shown in panel B. Each bar represents mean ± SEM HPASMC PTEN (A) mRNA or (B) protein levels. *P< 0.01 vs Normoxia (-), ^#^P< 0.01 vs Hypoxia (-) (n = 3–4).

In a previous report, our lab demonstrated that RSG attenuated hypoxia-induced PH, RVH, and pulmonary vascular remodeling, as well as hypoxic increases in platelet-derived growth factor receptor (PDGFR)β and hypoxic reductions in PTEN in the mouse lung[6]. RSG also attenuated hypoxia-induced PASMC proliferation in vitro[22]. These findings prompted the investigation of the effects of RSG on PTEN expression in hypoxia-exposed HPASMC. As shown in Fig 1A and 1B, hypoxia caused a significant reduction in PTEN mRNA and protein, and PTEN levels were restored in cells treated with RSG. These findings identify PTEN as an important target that RSG modulates to confer antiproliferative signals in hypoxia-exposed HPASMC.

### Hypoxia enhances miR-21 levels and reduces expression of its target PTEN

**Fig 2:**
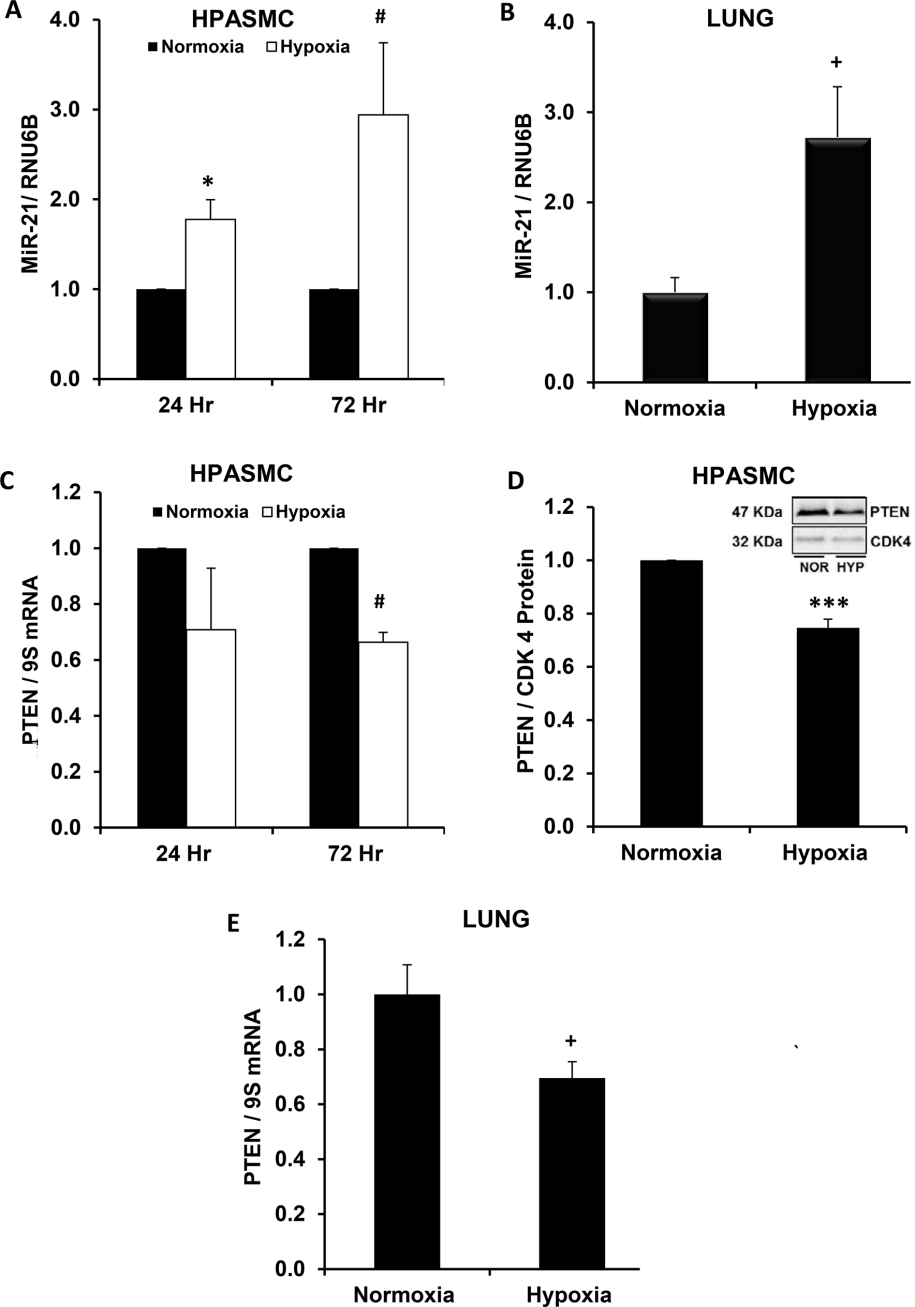
Hypoxia increases miR-21 and reduces PTEN levels in HPASMC in vitro and in the mouse lung in vivo. HPASMC were exposed to normoxic or hypoxic conditions for 24–72 hours (A,C,D) and mice were exposed to normoxia (21% O_2_) or hypoxia (10% O_2_) for 3 weeks (B,E). Large and small mRNA species were isolated from HPASMC and mouse lung lysates, and miR-21 and PTEN levels were quantified. A representative immunoblot is shown in panel D. Each bar represents mean ± SEM miR-21 or PTEN levels. *P< 0.05 vs Normoxia-24 Hr, ^#^P< 0.05 vs Normoxia-72 Hr, ^+^P< 0.05 vs Normoxia (Lung), ***P< 0.0001 vs Normoxia (HPASMC). A. (n = 6, 24 hr; n = 10, 72 hr); B. (n = 3); C,D. (n = 6); E. (n = 8).

To define associations between hypoxia, miR-21 and its target, PTEN, we examined the expression patterns of these cellular mediators in hypoxia-exposed HPASMC in vitro and in the hypoxic mouse lung in vivo. As illustrated in Fig 2A and 2B, hypoxia increased miR-21 expression in HPASMC and in the mouse lung, respectively. Hypoxia-induced miR-21 expression was associated with concurrent reductions its target PTEN (Fig 2C and 2E) in these models. These results demonstrate that patterns of miR-21 and PTEN expression in the hypoxic mouse lung parallel those occurring in hypoxia-exposed HPASMC, suggesting that these hypoxia-induced alterations are conserved between species and cell types.

### Overexpression of mature miR-21 mimic reduces PTEN levels and enhances HPASMC proliferation

**Fig 3:**
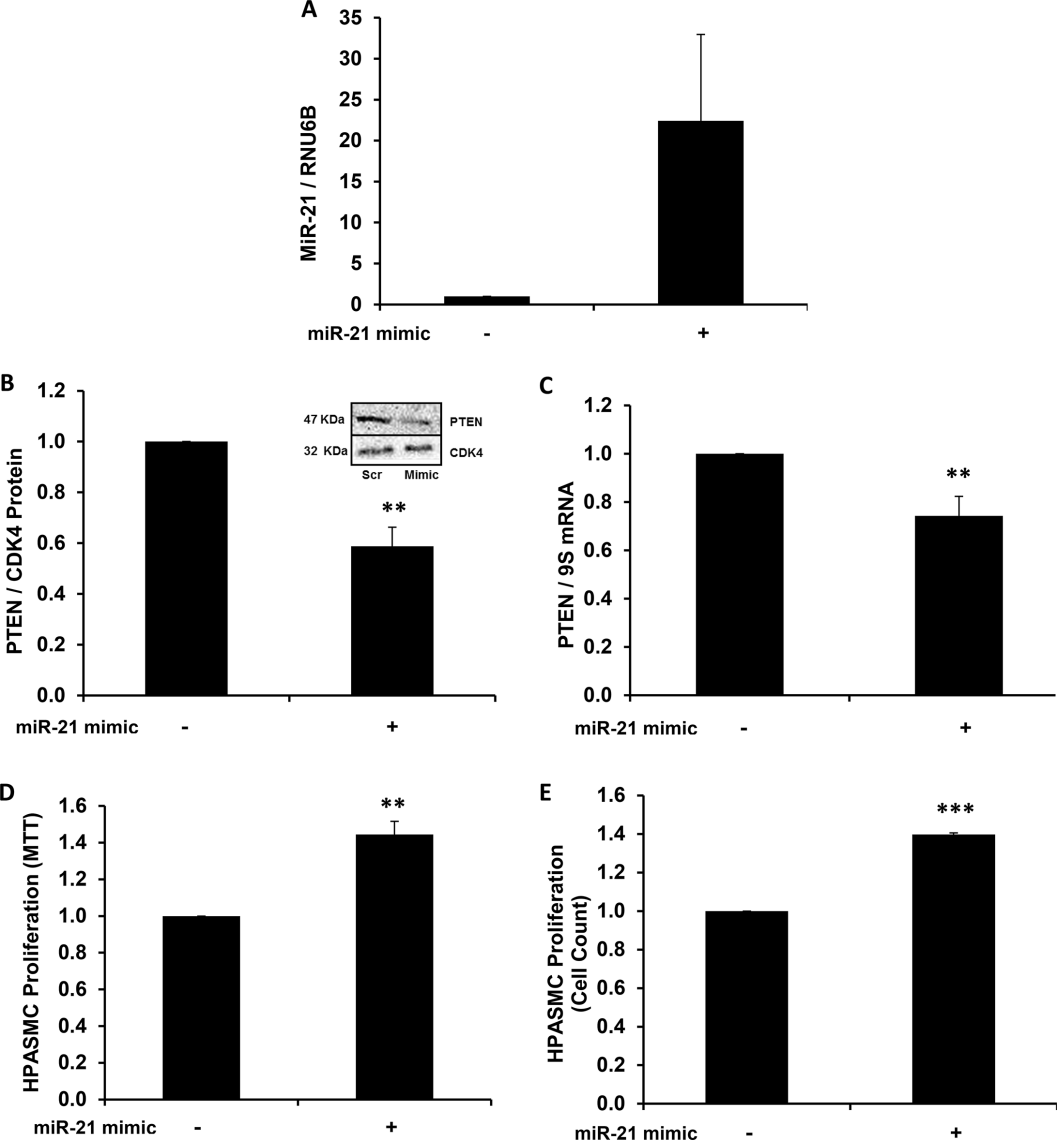
MiR-21 overexpression reduces PTEN levels and enhances normoxic HPASMC proliferation. (A-C) HPASMC were transfected with mature miR-21 mimic, and PTEN expression was analyzed. A representative immunoblot is shown in panel B. After transfection, HPASMC proliferation was measured by (D) MTT assay and (E) manual cell counting. Each bar represents mean ± SEM (A) miR-21 levels (B) PTEN protein, (C) PTEN mRNA, (D, E) proliferation. **P<0.01 vs Scrambled, ***P< 0.001 vs Scrambled. A,B,D,E. (n = 3–4); C. (n = 7).

Transfecting HPASMC with the synthetic mature miR-21 mimic enhanced miR-21 levels (Fig 3A) and significantly reduced PTEN protein (Fig 3B) and mRNA (Fig 3C) levels in HPASMC. To confirm functional effects of miR-21 overexpression on HPASMC proliferation, complementary techniques were employed to assess HPASMC proliferation following transfection with mature miR-21 mimic. Compared to HPASMC transfected with scrambled RNA sequences, mature miR-21 mimic-transfected HPASMC had increased proliferation compared to control, as measured by MTT assay (Fig 3D) and manual cell counting (Fig 3E).

### Depletion of miR-21 attenuates hypoxia-induced HPASMC proliferation

**Fig 4:**
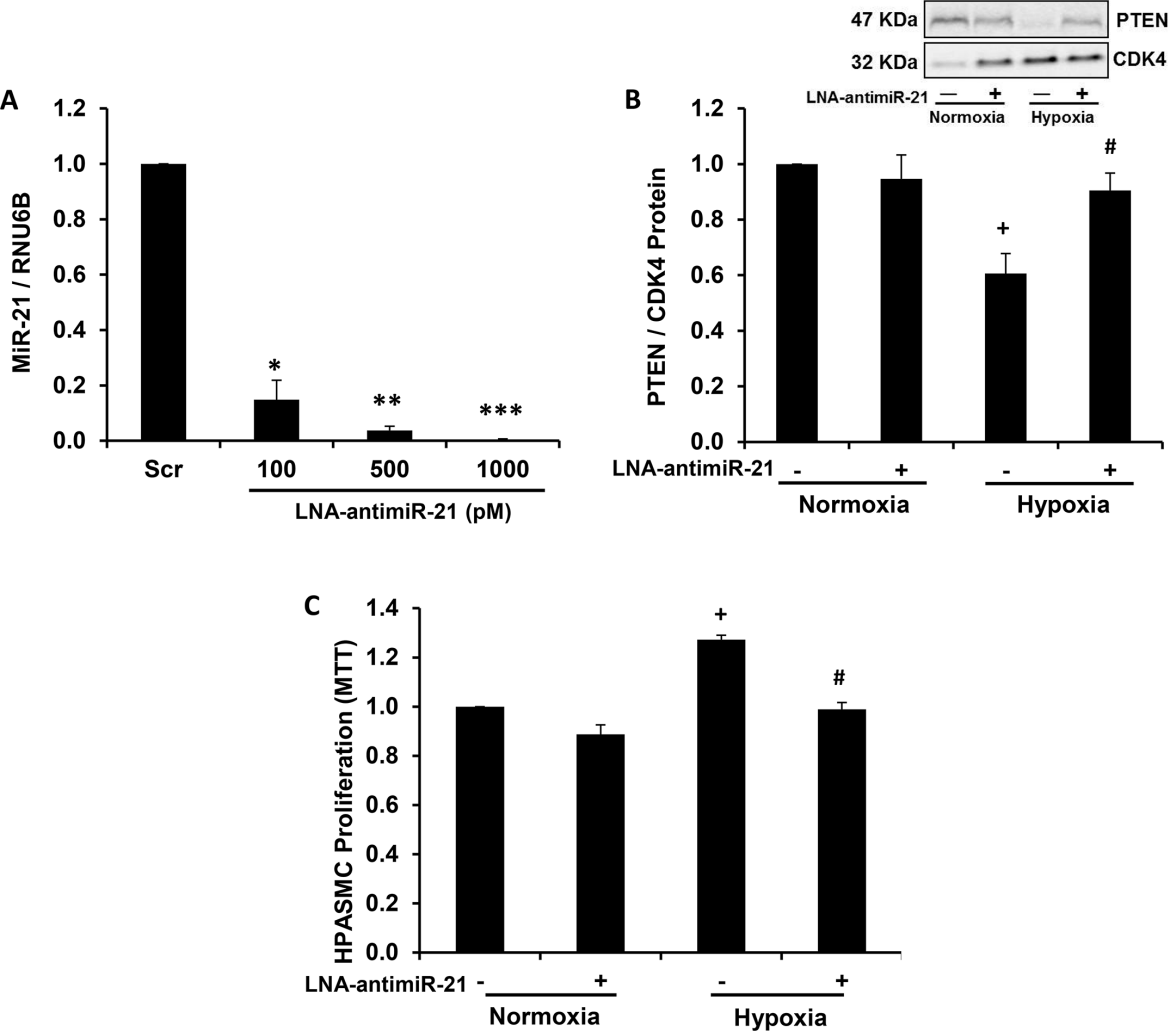
Depletion of miR-21 attenuates hypoxia-induced HPASMC proliferation. HPASMC were transfected with locked nucleic acid (LNA)-scrambled sequences or LNA-antimiR-21 (100–1000 pM), and (A) miR-21 levels were assessed. (B) PTEN protein, and (C) proliferation (MTT) were measured following LNA-antimiR-21 (1 nM) transfection. A representative immunoblot is shown in panel B. Each bar represents mean ± SEM miR-21 or PTEN expression or proliferation. *P< 0.05 vs Scr, **P< 0.001 vs Scr, ***P< 0.0001 vs Scr, ^+^P< 0.05 vs Normoxia (-), ^#^P< 0.05 vs Hypoxia (-). A,C. (n = 3–4), B. (n = 6).

To further confirm the importance of miR-21 in hypoxic HPASMC proliferation, HPASMC miR-21 levels were depleted by transfecting cells with miR-21 locked nucleic acid antisense oligonucleotide (LNA-antimiR-21). As shown in Fig 4A, transfection with graded concentrations of LNA-antimiR-21 induced stepwise reductions in HPASMC miR-21 levels, and miR-21 depletion attenuated hypoxic reductions in PTEN protein levels (Fig 4B). As shown in Fig 4C, HPASMC proliferation was significantly increased in hypoxia-exposed HPASMC, and transfection with LNA-antimiR-21 significantly attenuated hypoxia-induced HPASMC proliferation. These findings demonstrate that miR-21 plays a key functional role in proliferative responses of hypoxia-exposed HPASMC.

### Hypoxia-induced increases in miR-21 expression are attenuated by the administration of RSG

**Fig 5:**
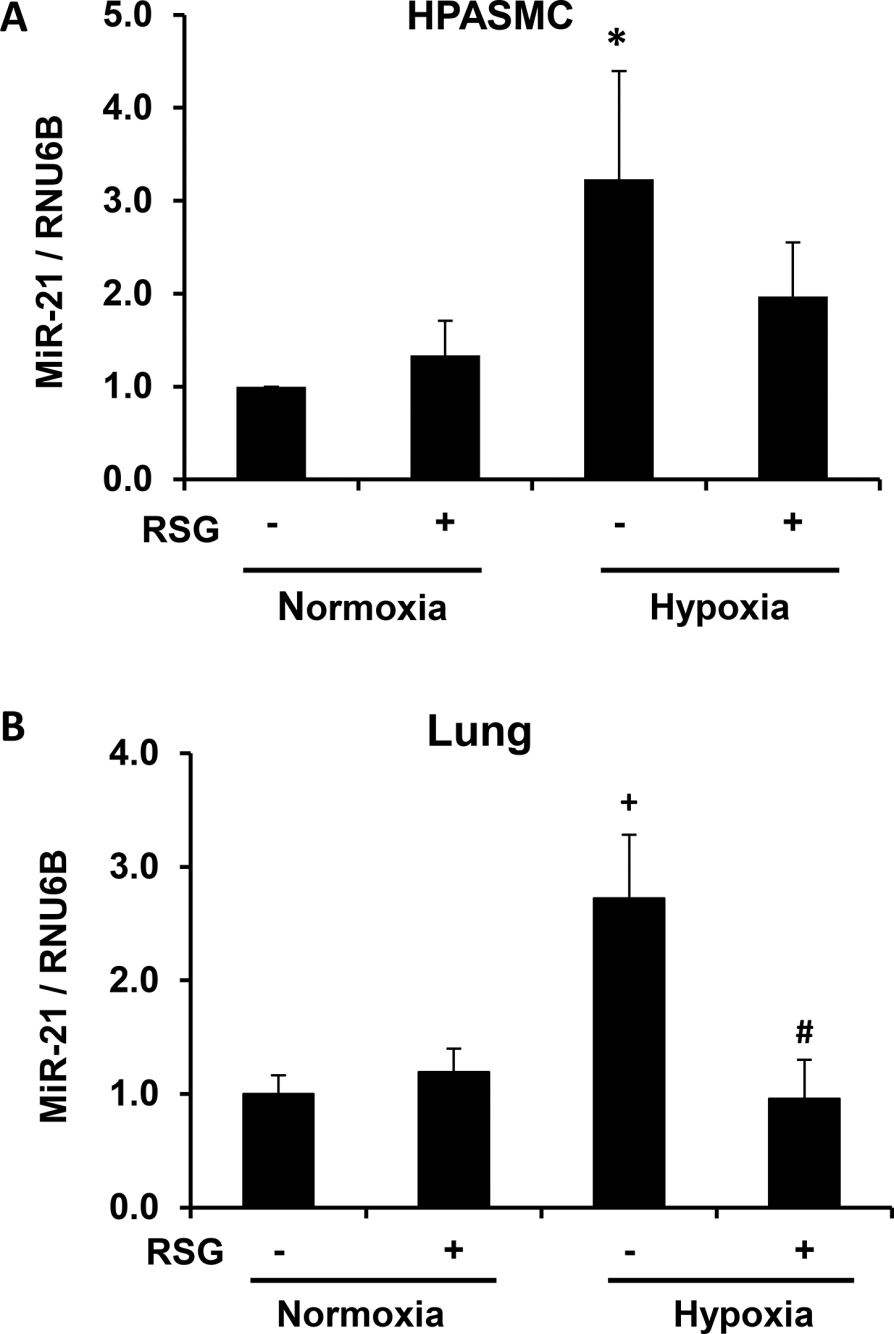
Rosiglitazone attenuates hypoxia-induced increases in miR-21 levels. (A) Hypoxia-exposed HPASMC were treated with RSG (10 μM) during the last 24 hours of the 72 hour study period. (B) RSG (10 mg/kg/day) was administered to mice daily during the last 10 days of the 3 week hypoxia exposure period, and miR-21 expression was quantified. Bars represent mean ± SEM miR-21 levels. *P< 0.05 vs Normoxia (-), ^+^P< 0.01 vs Normoxia (-), ^#^P< 0.01 vs Hypoxia (-) (n = 5–8).

RSG exerts several actions in PH by favorably altering the balance of multiple hypoxia-responsive genes that contribute to vascular cell proliferation[5, 6, 21, 22]. For example, we previously reported that RSG attenuated hypoxia-induced reductions in PTEN protein in the mouse lung in vivo[6]. Mechanisms through which RSG modulates PTEN expression in the mouse lung and HPASMC are not clearly defined. Therefore, we examined signaling interactions between RSG and miR-21. As shown in Fig 5A and 5B, miR-21 expression was increased in hypoxia-exposed HPASMC and in the hypoxic mouse lung and attenuated by treatment with RSG.

### RSG does not inhibit HPASMC proliferation induced by PTEN depletion

**Fig 6:**
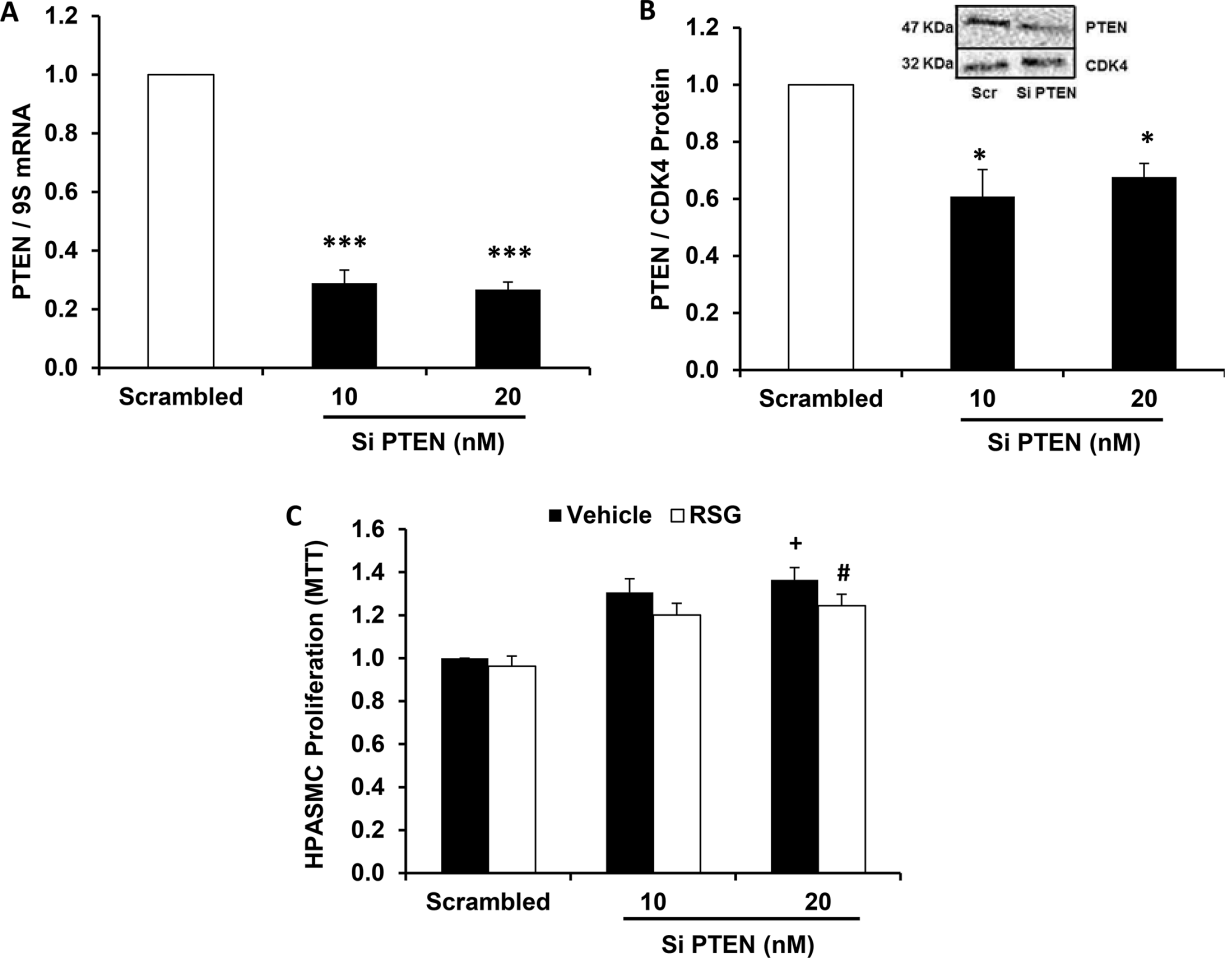
Rosiglitazone does not attenuate HPASMC proliferation in the absence of PTEN. HPASMC were transfected with PTEN small interfering RNA constructs. (A) PTEN mRNA and (B) PTEN protein levels were examined after transfection with PTEN siRNA. A representative immunoblot can be found in panel B. (C) HPASMC proliferation was assessed in PTEN-depleted HPASMC. In selected groups, RSG (10 μM) was added during the last 24 hours of the 72-hour incubation period prior to conducting the MTT proliferation assay. Each bar represents mean ± SEM HPASMC (A) PTEN mRNA, (B) PTEN protein, or (C) proliferation. *P< 0.05 vs Scrambled, ***P< 0.0001 vs Scrambled, ^+^P< 0.01 vs Scrambled-Vehicle, ^#^P< 0.05 vs Scrambled-RSG (n = 3–4).

To support our hypothesis that reductions in PTEN play a critical role in HPASMC proliferation, we depleted PTEN to determine its role in the regulation of proliferative signals in hypoxia-exposed HPASMC. As shown in Fig 6A and 6B, an approach that employed small interfering RNA was used to knockdown PTEN mRNA and protein to examine the functional effects of PTEN depletion on HPASMC proliferation. As shown in Fig 6C, increasing concentrations of siPTEN caused graded increases in basal HPASMC proliferation at 72 hours. In selected samples, the administration of RSG during the last 24 hours of incubation failed to attenuate proliferation. These results demonstrate that PTEN is a key antiproliferative mediator in HPASMC whose depletion is sufficient to enhance basal proliferation. The addition of RSG in identical concentrations to those that attenuated hypoxia-induced HPASMC proliferation[2, 22] failed to reduce proliferation induced by PTEN depletion. This finding highlights the importance of PTEN as a critical mediator through which RSG signals to inhibit cellular proliferation.

### Rosiglitazone attenuates hypoxia-induced increases in lung TGF-β1

**Fig 7:**
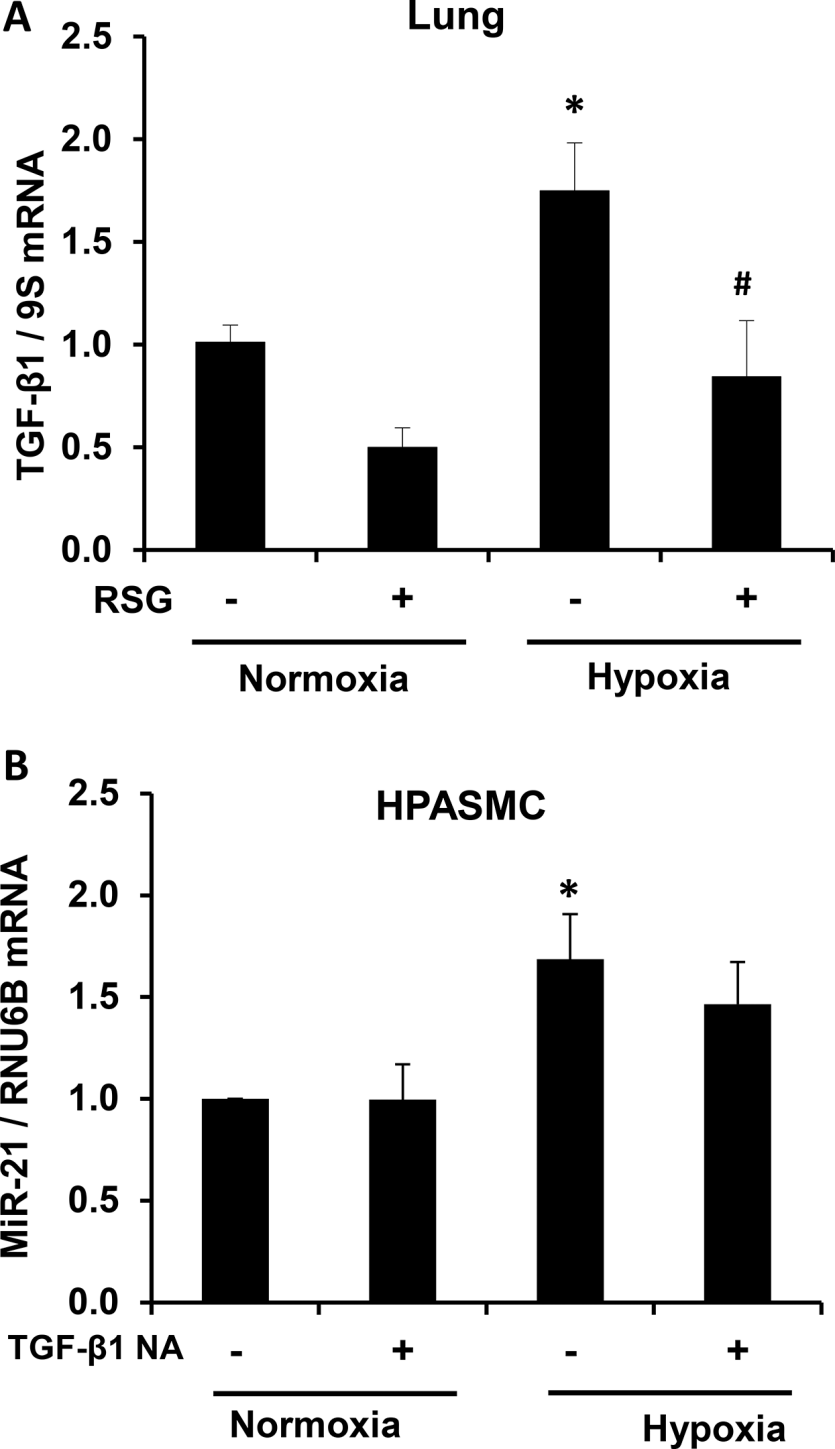
Rosiglitazone attenuates hypoxia-induced TGF-β1 expression. (A) Mice were exposed to normoxia or hypoxia for 3 weeks and treated with vehicle or RSG during the last 10 days, and (A) TGF-β1 mRNA was quantified. (B) HPASMC pre-treated with TGF-β1 neutralizing antibodies (1 μg/ml) for four hours were placed into a hypoxia chamber for 48 hours and miR-21 expression was quantified. Each bar represents mean ± SEM (A) TGF-β1 mRNA or (B) miR-21 levels. *P< 0.05 vs Normoxia (-), ^#^P< 0.01 vs Hypoxia (-) (n = 5–7)

Although little is known about the transcriptional regulation of miR-21, recent evidence demonstrates that TGF-β1 or BMP-associated SMAD signaling increases the accumulation of mature miR-21 through post-transcriptional mechanisms[33]. We measured TGF-β1 message levels in the hypoxic mouse lung to determine if RSG modulated its expression. As shown in Fig 7A, RSG attenuated chronic hypoxia-induced increases in TGF-β1 mRNA expression. To determine if TGF-β1 acts as an upstream mediator of miR-21 in our in vitro hypoxia model, we administered TGF-β1 neutralizing antibodies to HPASMC monolayers prior to exposure to hypoxia and examined miR-21 levels. As shown in Fig 7B, TGF-β1 neutralizing antibodies tended to attenuate hypoxic increases in HPASMC miR-21 levels. This finding demonstrates that RSG indirectly modulates miR-21 expression, in part, through upstream inhibitory effects on TGF-β1.

## Discussion

PH is a complex disorder that is phenotypically characterized by dysregulated proliferation of pulmonary vascular wall cells in conjunction with other well-described vascular insults[39–41]. This process narrows the vessel lumen cross-sectional area and progressively increases the pulmonary vascular resistance, leading to cor pulmonale and ultimately death. Our group previously demonstrated that administration of RSG attenuated hypoxia-induced HPASMC proliferation[2, 22] and both prevented and reversed established PH in the hypoxic mouse lung[6]. In this study, treatment regimens with RSG that produced favorable changes in pulmonary hemodynamics[6] were associated with increased lung PTEN expression. We extended these observations by identifying the role of miR-21 as a key posttranscriptional signaling mediator through which RSG increases PTEN to inhibit proliferation in hypoxia-exposed HPASMC. We demonstrated for the first time that administration of RSG in identical concentrations previously shown to attenuate HPASMC proliferation and PH concomitantly blunted hypoxia-induced increases in HPASMC miR-21 levels and abrogated hypoxic reductions in PTEN expression. Notably, inhibitory effects of RSG on HPASMC proliferation were lost following siRNA-mediated PTEN depletion. Furthermore, we demonstrated that mature miR-21 overexpression or PTEN depletion enhanced basal HPASMC proliferation and that miR-21 knockdown tended to rescue hypoxic reductions in PTEN and inhibited HPASMC proliferation. Lastly, RSG abrogated hypoxic increases in TGF-β1, an upstream mediator that is increased in PH and that increases miR-21 levels.

Because miR-21 is a well-established mediator of cellular growth and proliferation in vascular SMC[31, 34, 36], and its target gene, PTEN is implicated in PH pathogenesis[6, 26, 27], we hypothesized that miR-21 was a central mediator of HPASMC proliferation in response to hypoxia, and that RSG modulated miR-21 expression to confer antiproliferative signals in HPASMC. Our main study findings support this postulate and additionally confirm that RSG restores PTEN levels in part through inhibition of hypoxia-induced miR-21 expression in HPASMC and in the hypoxic mouse lung (Fig 5A and 5B).

A growing body of literature identifies miRs as key mediators of pathogenesis in pulmonary vascular disease and PH[29, 32, 42–46]. Importantly, recent reports suggested a possible role for miR-21 in human PH by demonstrating enhanced miR-21 levels in plexiform and concentric obliterative vascular lesions[47] and in the lung tissue of patients with PAH[32]. MiR-21 levels are also elevated in the lung tissue and small pulmonary artery profiles of rodents in whom PH was induced experimentally[31, 32]. In experimental models of PH, miR-21 depletion is associated with hemodynamic, vascular or RV morphometric improvements [31, 35]. MiR-21 stimulates the growth of VSMC, both by increasing the expression of cell cycle regulators and mediators of proliferation, including Cyclin D1[31], Bcl-2[36], and PCNA[31, 34] and by suppressing levels of antiproliferative or proapoptotic mediators such as PDCD4[34, 48], SPRY[34], and PTEN[36]. The current study linked miR-21 expression with reductions in PTEN in hypoxia-exposed HPASMC and identified miR-21 as a mediator through which RSG modulates PTEN.

As shown in Fig 1A and 1B, RSG attenuated hypoxia-induced reductions in HPASMC PTEN expression. However, in Fig 6C, RSG did not inhibit HPASMC proliferation induced by PTEN depletion. These findings support the role of PTEN as a key mediator through which RSG confers antiproliferative signals. A likely mechanism through which RSG increases PTEN occurs through inhibition of hypoxic increases in miR-21 levels (Fig 5A and 5B). MiR-21 has previously been targeted by antagomirs to attenuate experimental PH[31, 35], but to our knowledge, the current study is the first to successfully employ a pharmacological ligand (RSG) to inhibit miR-21. Few previous reports have described interactions between PPARγ and miR-21. For example, in diabetics with hypertension, pioglitazone increased circulating miR-21 levels and brachial artery flow-mediated dilation [49]. These findings differ from our observations that PPARγ ligands attenuate miR-21 levels. However, because the correlation between circulating and tissue miR levels has not been firmly established, and because miR expression is often tissue- and stimulus-specific, comparisons of miR expression between the systemic and pulmonary circulations must be undertaken with caution.

PPARγ activity and expression can be modulated through a variety of mechanisms that involve transcriptional inhibition, posttranslational modification, and protein-protein interactions. Emerging data demonstrate that PPARγ can be inhibited through posttranscriptional mechanisms that involve miRs [50–52]. The current study examines the ability of PPARγ to alter the posttranscriptional expression of target mRNA species through miR signaling. Since RSG belongs to the well-studied thiazolidinedione class of PPARγ activating drugs, the clinical relevance of our findings should encourage the use this existing and approved pharmaceutical to study non-coding RNA in PH, which may speed translation of novel therapeutic approaches.

PPARγ, as a ligand-activated transcription factor, alters the transcriptional expression of target genes by binding to PPAR response elements (PPRE) in their promoters. PTEN hosts 2 PPRE in its promoter region that when activated modulate its transcriptional expression [53]. However, as demonstrated in Fig 1A and 1B, RSG did not increase basal PTEN expression under normoxic conditions whereas it significantly attenuated hypoxia-induced reductions in PTEN. These findings indicate that PPARγ activation plays a minor role in PTEN expression under normoxic conditions, but that PPARγ activation can reverse hypoxic reductions in PTEN. These findings led us to postulate that PPARγ regulates PTEN by modulating miR-21 levels rather than direct transcriptional activation of the PTEN promoter. The results in Fig 5A and 5B support this postulate by demonstrating that RSG attenuated hypoxia-induced increases in miR-21 expression while having no significant effect on miR-21 levels in normoxic cells. As shown in Fig 4B, depletion of miR-21 similarly rescued hypoxia-induced reductions in PTEN. However, neither administration of RSG nor depletion of miR-21 altered the constitutive expression of HPASMC PTEN in normoxia-exposed HPASMC. Taken together, our findings suggest that miR-21 plays a relatively minor role in regulating constitutive PTEN levels in normoxic cells. However, under hypoxic conditions, when miR-21 levels are increased, this post-transcriptional pathway plays a more prominent role that reduces PTEN levels.

As recently reported, activation of PPARγ with RSG decreased the transcriptional activity of AP-1 and NFκB in endothelial cells[54]. Since both AP-1[55] and NFκB[28] stimulate miR-21 transcription by binding to conserved sites in the miR-21 promoter, and RSG decreases the transcriptional activity of AP-1 and NFκB[54], it is plausible that in our model RSG attenuates hypoxia-induced miR-21 transcription through regulation of these transcription factors. PPARγ agonists modulate a variety of vascular mediators involved in PH pathogenesis as previously reviewed[3, 4, 56]. Since TGF-β1 is involved in PH pathogenesis, and repressive interactions have been described between PPARγ and TGF-β1 in alveolar epithelial cells[57], we examined this relationship in the context of miR-21 signaling in the mouse lung.

As demonstrated by our findings in Fig 7A, RSG attenuated hypoxia-induced TGF-β1 expression in the mouse lung. In concert with our findings that RSG attenuated hypoxia-induced miR-21 expression (Fig 5A and 5B), this result suggests that the inhibitory effects of RSG on miR-21 are mediated through TGF-β1. Fig 7B supports this postulate by demonstrating that TGF-β1 neutralization decreased miR-21 levels in hypoxia-exposed HPASMC. Collectively, our findings confirm that TGF-β1 is an important target through which PPARγ agonists modulate miR-21 expression. The role of transcription factors such as AP-1 and NFκB in the regulation of miR-21 by RSG is not defined in the current study, but merits additional consideration in future investigations.

The potential for miR-21 to serve as a therapeutic target in human PH is underscored by emerging literature that links depletion of the miR-21 target, PTEN, with sequelae of enhanced SMC proliferation, including PH. For example, Pi, et.al, demonstrated that PTEN inhibition reversed antiproliferative effects of BMP-2 administration in HPASMC in vitro[58]. Both monocrotaline and chronic hypoxia models of experimental PH were associated with reduced lung PTEN expression and vascular remodeling[26], and mice with selective PTEN depletion in SMC developed irreversible PH, vascular remodeling, and complex vascular lesions in hypoxic environments[27]. These studies highlight the importance of PTEN in the maintenance of balanced SMC growth and vascular homeostasis. Finally, human lung tissue specimens from patients with PAH exhibited enhanced PTEN inactivation in comparison to normotensive control patients. Collectively, these reports demonstrate that the proapoptotic and antiproliferative features of PTEN are conserved among species and function similarly in a heterogeneous variety of tissue types. Furthermore, the ability of RSG to increase PTEN levels through direct transcriptional activation or posttranscriptionally through inhibition of miR-21 highlights the unique and varied mechanisms of action of pharmacological activators of PPARγ.

A recognized limitation of the current study is the isolated examination of the contribution of a single miR to PH pathogenesis. While system-based approaches more effectively capture the complexity and interconnectedness of miR signaling in PH[32, 59], paradigm shifting observations have been made by individual investigations of miRs including miR-204[43], -17–92[45], and -143/145[60]. We also recognize that because PPARγ ligands simultaneously modulate multiple major pathologic vascular derangements in PH[3, 4], their therapeutic actions likely occur through both miR-dependent and miR-independent pathways. Finally, cardiovascular safety concerns have been raised regarding the use of rosiglitazone in diabetic patients [61], and pioglitazone may increase the risk of bladder cancer[62]. These reports highlight the need for longitudinal studies with observational follow up and emphasize the importance of more thoroughly defining mechanisms by which individual PPARγ ligands exert their pharmacological effects.

**Fig 8:**
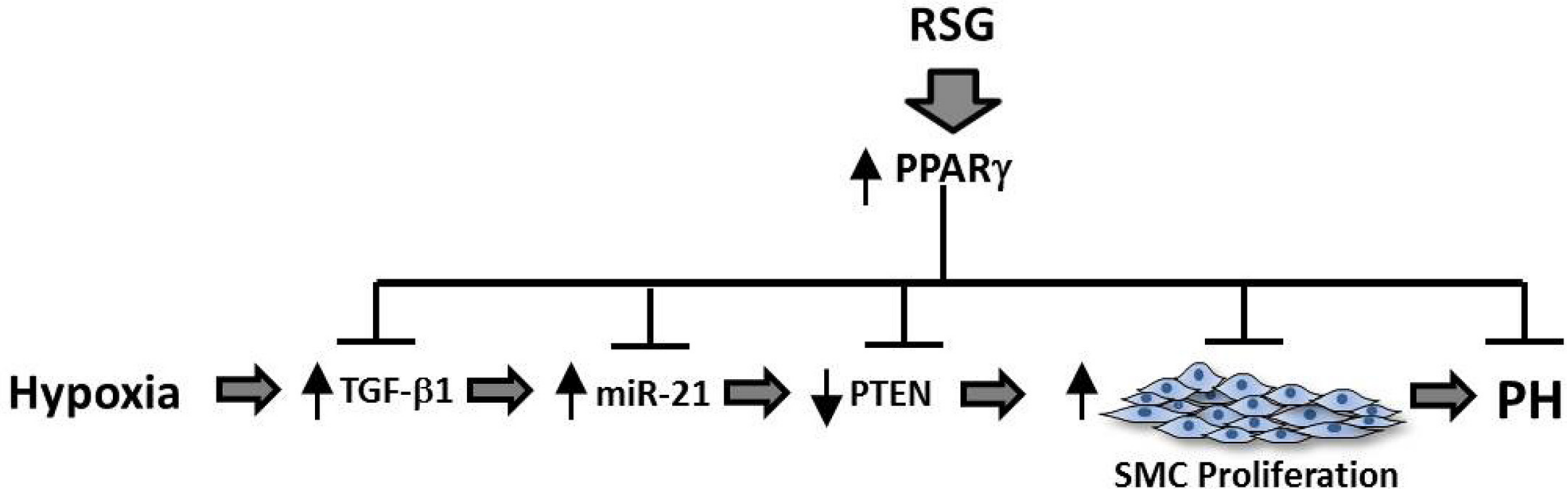
Hypoxia-induced alterations in miR-21 and PTEN that mediate proliferative responses of HPASMC to hypoxia are modulated by RSG. The schema depicts the proposed signaling interactions involved in hypoxia-induced vascular SMC proliferation. Hypoxia exerts mitogenic effects on HPASMC through stimulation of miR-21 which and reciprocally reduces PTEN. TGF-β1 is a recognized mediator of HPASMC proliferation that may exert effects upstream of miR-21. Inhibitory actions of the PPARγ ligands are due to RSG effects on multiple mediators involved in proliferative responses to hypoxia.

As illustrated by the schema depicted in Fig 8, we conclude that miR-21-mediated reductions in PTEN play a central role in HPASMC proliferative responses to hypoxia and that RSG confers antiproliferative effects by preventing hypoxic increases in miR-21, thereby deprepressing PTEN. Ongoing studies are designed to define how RSG modulates miR-21 expression and to identify additional targets regulated by miR-21 that effect PASMC proliferation in response to hypoxia. This line of investigation provides additional clarification of molecular mechanisms underlying the therapeutic effects of PPARγ ligands in PH. These results can promote the discovery and development of novel therapeutic approaches for PH.

## Supporting Information

S1 Fig Arrive Guidelines Checklist. (DOCX)

S2 Fig Data set. (XLSX)
