# Supplementary material for: PPARγ Ligands Attenuate Hypoxia-Induced Proliferation in Human Pulmonary Artery Smooth Muscle Cells through Modulation of MicroRNA-21
Source: PLoS One. 2015 Jul 24;10(7):e0133391. doi: 10.1371/journal.pone.0133391 (PMC4514882; doi:10.1371/journal.pone.0133391)
Supplement: S1 Fig — (DOCX) [file pone.0133391.s001.docx]

**The ARRIVE Guidelines Checklist**

|Item| |Recommendation| |Section/Paragraph|

1. Title: PPARγ ligands attenuate hypoxia-induced proliferation in human pulmonary artery smooth muscle cells through modulation of microRNA-21

2. Abstract: Abstract

**Introduction**

3. Background: Introduction (¶ 2-4), Discussion (¶ 3,8)

4. Objectives: We hypothesized that hypoxic increases in miR-21 suppress PTEN and promote PASMC proliferation and that PPARγ activation, by attenuating hypoxia-induced increases in miR-21 expression, reduces proliferation. Introduction (¶ 4)

**Methods**

5. Ethical Statement: All scientific investigations and procedures that involved animals were reviewed and formally approved by the Atlanta Veterans Affairs Medical Center Institutional Animal Care and Use Committee.

Materials and Methods (¶ 1)

6. Study Design: The main study objective is to examine the role of miR-21 in the regulation of proliferative pathways involved in hypoxia-induced PH and to determine if PPARγ ligands attenuate PH by modulating miR-21 expression. Our in vivo model employs 4 experimental groups, which contain 3-5 mice per group. Mice in the control group are exposed to normoxia and treated with vehicle, methylcellulose. In the remaining experimental groups, mice are exposed to normoxia + rosiglitazone (RSG), or hypoxia + RSG or methylcellulose. To minimize subjective bias, mice were randomly assigned to control or treatment groups. Additionally, mice were housed in normoxic or hypoxic environments for identical periods and equivalent volumes of methylcellulose or RSG were administered to mice in respective control or treatment groups.

Materials and Methods (¶ 1)

7. Experimental Procedures: Mice are housed in cages at room air (21% oxygen) or in a hypoxia chamber where they breathe air containing 10% oxygen to simulate the conditions of oxidative stress that promote pulmonary hypertension. Mice are caged in normoxic or hypoxic environments for 3 weeks. The hypoxia chamber is the established model for studying pulmonary hypertension in mice. This model has been well documented in the literature, and shows no ill effects upon mouse comfort. Drug intervention with the PPARγ activator, RSG (10 mg/kg, 100 μl) or an equal volume of methylcellulose is administered daily via oral gavage for the last 10 days of the study period. This dose was chosen because previous studies from our lab demonstrated that hypoxia-induced pulmonary hypertension, right ventricular hypertrophy and vascular remodeling were attenuated at this drug dose. Oral gavage was chosen as the preferred method of medication delivery because it is a minimally invasive technique that does not cause undue pain or distress. Animals were euthanized as part of the planned studies upon the conclusion of the experiment. Animals were euthanized by insufflating CO_2_ from a compressed gas tank into a clear plastic chamber in which they were housed for 3-4 minutes. Death was primarily verified by physical examination detecting a prolonged absence of spontaneous respirations, and secondarily by thoracotomy with direct visualization and removal of the heart and lungs.

Materials and Methods (¶ 1)

8. Experimental Animals: All animals used in the current study were male C57/BL6J mice obtained from Jackson Laboratories. Mice were 8-10 weeks old and weighed approximately 25 gm. All animals were SPF and drug-naive.

Materials and Methods (¶ 1)

9. Housing and Husbandry: All mice used for our studies are housed in an SPF animal facility on site at the Atlanta Veterans Affairs Medical Center in microisolator cages. Between 3-5 animals are housed jointly per cage. In accordance with facility protocol, light and dark cycles rotate every 12 hours, cage bedding is changed twice weekly and food and water are available ad-libitum. Specific behaviors such as labored breathing, inactivity, sunken eyes, or piloerection are monitored and brought to the attention of veterinary staff if detected. Emory endpoint guidelines are followed. Animal weights are monitored every 2-weeks until the end of the study period.

10. Sample Size: A total of 48 mice were used in the current study. Between 3-5 mice were used in each of the 4 experimental groups for each study. The number of mice needed for the study was determined using a power analysis based on preliminary data drawn from a prototypical study featuring the same experimental groups that was powered to detect an overall difference among the group means using an alpha of 0.05.

11. Allocating Animals to Experimental Groups: Mice are randomly assigned to the aformentioned experimental groups. Upon arrival, mice are initially inventoried in our animal facility. Mice are assigned an identification code based on their strain, generation, and number of siblings. To differentiate between mice in the same cage, ears are punched using a combination of right and left ear punches with no mouse receiving more than two punches. The animals are monitored after ear-punches to ensure they are not exhibiting signs of distress. At the onset of the chronic hypoxia exposure studies, identical numbers of mice in different treatment groups are maintained in their respective cages and placed in hypoxic or normoxic environments for 3 weeks ± treatment with RSG.

12. Experimental Outcomes: Primary outcomes were to examine the effects of hypoxia, miR-21, and PPARγ activation on human pulmonary artery smooth muscle cell proliferation. Secondary outcomes were to confirm and explore molecular mechanisms through which PPARγ ligands attenuate hypoxia-induced increases in miR-21 expression in the mouse lung and in HPASMC.

Discussion (¶ 1)

13. Statistical Methods: For each of the proposed studies, a one-way ANOVA and an alpha of 0.05 was used to determine overall differences between sub-groups. In groups that significantly differed, all pairwise comparisons were tested using Tukey’s method for multiple comparisons to determine the nature of this difference. The plan for statistical analysis assumed normal distribution of data. Differences in groups featuring experiments with 2 variables were detected using 2-tailed unpaired t-tests.

Materials and Methods (¶ 7)

**Results**

14. Baseline Data: Mice in each experimental group tested were males between 8-10 weeks of age. Mice weighed approximately 25 gm and were SPF and drug-naive.

15. Numbers Analyzed: Data from 45/48 animals were included in the analysis. Normoxia (n=12), Normoxia + RSG (n=11), Hypoxia (n=11), Hypoxia + RSG (n=11). Mice not included in the analysis were statistical outliers as defined by Grubbs’ test.

16. Outcomes and estimation: Results (¶ 1-7)

17. Adverse Events: No adverse events occurred.

**Discussion**

18. Interpretation / Scientific Implications: Discussion (¶ 4,5,7-9)

19. Generalizability / Translation: Discussion (¶ 3, 4, 8)

20. Funding:

VA Career Development Award (1lK2 BX001707-01A1) David Green-PI

Merit Review Award (101BX001910-01) C. Michael Hart-PI

National Institutes of Health (R01 HL102167) C. Michael Hart, Roy. L. Sutliff - Co-PI
